# Transmission dynamics of *SARS-CoV-2* variants in the Brazilian state of Pará

**DOI:** 10.3389/fpubh.2023.1186463

**Published:** 2023-07-05

**Authors:** Catarina T. Pinho, Amanda F. Vidal, Tatianne Costa Negri Rocha, Renato R. M. Oliveira, Maria Clara da Costa Barros, Laura Closset, Jhully Azevedo-Pinheiro, Cíntia Braga-da-Silva, Caio Santos Silva, Leandro L. Magalhães, Pablo Diego do Carmo Pinto, Giordano Bruno Soares Souza, José Ricardo dos Santos Vieira, Rommel Mario Rodríguez Burbano, Maísa Silva de Sousa, Jorge Estefano Santana de Souza, Gisele Nunes, Moises Batista da Silva, Patrícia Fagundes da Costa, Claudio Guedes Salgado, Rita Catarina Medeiros Sousa, Wim Maurits Sylvain Degrave, Ândrea Ribeiro-dos-Santos, Guilherme Oliveira

**Affiliations:** ^1^Laboratório de Genética Humana e Médica, Instituto de Ciências Biológicas, Universidade Federal do Pará, Belém, Pará, Brazil; ^2^Instituto Tecnológico Vale, Belém, Pará, Brazil; ^3^Programa de Pós-Graduação em Genética e Biologia Molecular, Universidade Federal do Pará, Belém, Pará, Brazil; ^4^Laboratório de Biologia Molecular, Hospital Ophir Loyola, Belém, Pará, Brazil; ^5^Núcleo de Medicina Tropical, Universidade Federal do Pará, Belém, Pará, Brazil; ^6^Programa de Pós-Graduação em Bioinformática, Universidade Federal do Rio Grande do Norte, Natal, Rio Grande do Norte, Brazil; ^7^Bioinformatics Núcleo Multidisciplinar de Bioinformática, Universidade Federal do Rio Grande do Norte, Natal, Rio Grande do Norte, Brazil; ^8^Laboratório de Dermatologia e Imunologia, Instituto de Ciências Biológicas, Universidade Federal do Pará, Marituba, Pará, Brazil; ^9^Hospital Universitário João de Barros Barreto, Universidade Federal do Pará, Belém, Pará, Brazil; ^10^Laboratório de Genômica Funcional e Bioinformática, Instituto Oswaldo Cruz, Fundação Oswaldo Cruz, Rio de Janeiro, Brazil

**Keywords:** genomic surveillance, Brazil, *SARS-CoV-2*, COVID-19, Amazon, gamma

## Abstract

**Introduction:**

After three years since the beginning of the pandemic, the new coronavirus continues to raise several questions regarding its infectious process and host response. Several mutations occurred in different regions of the *SARS-CoV-2* genome, such as in the spike gene, causing the emergence of variants of concern and interest (VOCs and VOIs), of which some present higher transmissibility and virulence, especially among patients with previous comorbidities. It is essential to understand its spread dynamics to prevent and control new biological threats that may occur in the future. In this population_based retrospective observational study, we generated data and used public databases to understand *SARS-CoV-2* dynamics.

**Methods:**

We sequenced 1,003 *SARS-CoV-2* genomes from naso-oropharyngeal swabs and saliva samples from Pará from May 2020 to October 2022. To gather epidemiological data from Brazil and the world, we used FIOCRUZ and GISAID databases.

**Results:**

Regarding our samples, 496 (49.45%) were derived from female participants and 507 (50.55%) from male participants, and the average age was 43  years old. The Gamma variant presented the highest number of cases, with 290 (28.91%) cases, followed by delta with 53 (5.28%). Moreover, we found seven (0.69%) Omicron cases and 651 (64.9%) non-VOC cases. A significant association was observed between sex and the clinical condition (female, *p* = 8.65e-08; male, *p* = 0.008961) and age (*p* = 3.6e-10).

**Discussion:**

Although gamma had been officially identified only in December 2020/January 2021, we identified a gamma case from Belém (capital of Pará State) dated May 2020 and three other cases in October 2020. This indicates that this variant was circulating in the North region of Brazil several months before its formal identification and that Gamma demonstrated its actual transmission capacity only at the end of 2020. Furthermore, the public data analysis showed that *SARS-CoV-2* dispersion dynamics differed in Brazil as Gamma played an important role here, while most other countries reported a new infection caused by the Delta variant. The genetic and epidemiological information of this study reinforces the relevance of having a robust genomic surveillance service that allows better management of the pandemic and that provides efficient solutions to possible new disease-causing agents.

## 1. Introduction

In December 2019, an outbreak of pneumonia was reported in Wuhan, the capital of the Hubei province in China. The associated symptoms are referred to as coronavirus disease 2019 (COVID-19), and its rapid spread caused a global pandemic officially declared by the World Health Organization (WHO) on March 11, 2020. The COVID-19 pandemic, whose etiologic agent is the severe acute respiratory syndrome coronavirus 2 (*SARS-CoV-2*), has caused almost seven million worldwide deaths in the three years since its discovery in Wuhan (1, 2). To this date (may/2023), the Americas present more than 192.4 million cases, while Brazil has around 37.4 million cases (2).

*SARS-CoV-2* is a Betacoronavirus that belongs to the *Coronaviridae* family, which is constituted mainly by agents that cause the common cold and severe acute respiratory syndrome (SARS), such as *SARS-CoV-1* (3, 4). *SARS-CoV-2* has a 30-kb genome that encodes four major structural proteins: nucleocapsid (N), membrane (M), envelope proteins (E), and spike (S) (5). The spike protein mediates the virus entry into host cells, and once inside the cell, the virus replicates and may cause high levels of inflammation, which favors opportunistic infections (6, 7).

Clinical manifestations of COVID-19 are highly heterogeneous, even among patients with the same gender, age, and risk group. Some are asymptomatic, while others may present dry cough, fever, headache, fatigue, odynophagia, and diarrhea. Some patients present the most severe symptoms, such as pneumonia and thromboembolic events, which may lead them to death (8, 9).

The cause of the differential patterns of symptoms in COVID-19 remains unclear. Still, it seems to be explained by several factors, including the patient’s genetic background, previous comorbidities, and the *SARS-CoV-2* variants (8–10). These variants are classified according to the set of mutations in the viral genome. Since March 2020, several lineages of *SARS-CoV-2* have emerged, and those with higher virulence and ability to propagate were classified as variants of concern (VOCs), followed by the variants of interest (VOIs) and variants under monitoring (VUMs), in descending order of virulence. Five VOCs have been recognized: B.1.1.7 (U.K, Alpha variant), B.1.351 (South Africa, Beta variant), P.1 (Brazil, Gamma variant), B.1.617.2 (India, Delta variant), and B.1.1.529 (multiple countries, Omicron variant) (11).

Most mutations present in these variants are in the spike gene and enable a more effective entry of the virus into the host cells, resulting in both high viral load and levels of inflammation, which leads to an increased risk of developing the severe form of COVID-19 (12). Even though Omicron has a higher number of mutations, the symptoms caused by it were less severe when compared with the other lineages, probably due to the higher vaccine coverage of the world population at the time of its emergence (13–15).

VOCs have presented different patterns of prevalence across the globe. For instance, Gamma, which is a descendant of B.1.1.28, showed a high prevalence in Brazil and South America. Since the first Gamma case, in December 2020, FIOCRUZ database has reported 56.877 infections caused by Gamma. It is important to mention that these numbers are probably lower than reality, due to the notification missing data (16).

Gamma originated in Brazil’s Amazon region and displays 12 mutations in the *S* gene, three of them (K417T, E484K, and N501Y) in the receptor binding domain (RBD). These mutations potentially increase transmissibility and prevent virus immune recognition by the host (7, 11). This VOC was first identified in Brazil in December 2020, in the city of Manaus, capital of the Amazonas state, and since then until the end of 2021 it caused several cases in Brazil, especially in the North region (17).

According to Our World in Data,^1^ more than 765 million cases and more than 6.9 million deaths worldwide have been reported. In Brazil, there are estimated to be more than 36 million cases and about 695,000 deaths. Pará (PA) was the most affected by the pandemic in the North region of Brazil, with 861,000 cases and more than 18,000 deaths (18).

In this study, we provide data collected by a regional network for COVID-19 genomic surveillance in the state of Pará in the North of Brazil and map up the status of transmission dynamics of *SARS-CoV-2* during these three years of the pandemic of COVID-19 worldwide. Besides that, this study’s main aim is to help the scientific community, specially the Brazilian one, to understand the spreading of coronavirus variants in the North region of Brazil and also to help this community to prevent and control new biological threats that may occur in the future. Furthermore, this study may have significant contributions to the understanding of possible correlations between coronavirus variants and the host’s clinical conditions and comorbidities.

## 2. Materials and methods

### 2.1. Study design and sample collection

This population-based retrospective observational study uses genomic and COVID-19 surveillance data collected from the state of Pará in North Brazil. The data presented in this study result from a regional network headed by Instituto Tecnológico Vale (Belém/PA, Brazil) and Fundação Oswaldo Cruz (Fiocruz - Rio de Janeiro/RJ, Brazil), In which several institutions gathered efforts to accomplish a COVID-19 genomic surveillance in Brazil.^2^

From May 2020 to October 2022, we sequenced 1,003 naso-oropharyngeal swabs and saliva samples (136 saliva samples and 867 swab samples) from Pará from May 2020 to October 2022. The mean age was 43 years old. We included samples from 67 municipalities of Pará, but most are from Belém, its capital (Supplementary Table S1; Supplementary Figure S1). Swab samples were collected and transferred to viral transport media, and saliva samples were collected in sterile plastic collection tubes. After collection, both sample types were stored at −80°C until further analysis.

The study, including all experimental protocols, was approved by the Ethics Committee of the Center of the University Hospital João de Barros Barreto of the Federal University of Pará (No. 50865721.1.0000.0017). All study participants or their legal guardians provided informed written consent in accordance with the Helsinki Declaration. A summary of clinical data is presented in Table 1, and the detailed metadata is in Supplementary Table S1.

**Table 1:** Clinical characteristics of COVID-19 patients (asymptomatic, mild symptoms, and severe disease) versus variants (Alpha, Beta, Gamma, Delta, Omicron, and non-VOCs).

Clinical characteristics	Asymptomatic (*n* = 122)	Mild (*n* = 219)	Severe (*n* = 116)	*p*-value*
Sex, *n* (%)				
Female	50 (40.98%)	116 (52.96%)	62 (53.45%)	**8.65e-08**
Male	72 (59.02%)	103 (47.04%)	54 (46.55%)	**0.008961**
Age, mean (SD)				
	43.9 (16)	37.3 (17.8)	51.3 (18.2)	**3.6e-10**
Variant**s**				
Alpha	0	0	0	1
Beta	2 (1.63%)	0	0	0.1353
Gamma	57 (46.72%)	88 (40.18%)	58 (50%)	0.08798
Delta	12 (9.83%)	27 (12.33%)	6 (6.17%)	**0.01019**
Omicron	0	7 (3.20%)	0	**0.0009119**
Non-VOCs	51 (41.80%)	97 (44.30%)	52 (44.83%)	**3.183e-05**

SD, standard deviation. *p-values were calculated using Chi-squared test (for sex and variants) and Kruskal-Wallis test with correction for multiple comparisons (for age). Bold values are statistical significant.

### 2.2. RNA isolation, qRT-PCR, and sequencing

Viral RNA was isolated from naso-oropharyngeal swabs and saliva samples using MagMAX Viral/Pathogen Nucleic Acid Isolation Kit (Thermo Fisher Scientific) at KingFisher System (Thermo Fisher Scientific). These kits are designed to work with robots that can isolate genetic material from various sample types, pathogens, and hosts. To ensure a sterilized extraction environment, it is crucial to sanitize both kits before use and expose them to ultraviolet light (UV), if possible.

The Maxwell protocol is a straightforward process that involves adding around 200 μL of the sample (saliva or swab) to a microtube containing a solution of 200 μL and 20 μL of Proteinase K. The microtube is then vortexed briefly and incubated at 56°C for 10 min. While the sample is incubating, extraction cartridges, tips, and microtubes (0.6 mL) containing nuclease-free water are prepared and added to the robot. The robot then performs the extraction, which takes 42 min and yields 50 μL of sample. In contrast, the automated extraction performed by KingFisher can be more time-consuming and complex. It requires four 96-well plates, the first containing the samples, bead solution (binding solution and beads), and proteinase K, the second with alcohol 80%, the third with Wash Solution from the kit, and the fourth with the elution buffer. After preparing the plates with their respective amounts of samples and reagents, the plates are loaded onto the robot for automated extraction, which takes approximately 25 min and yields approximately 30 μL of sample.

Viral RNA was detected using TaqPath 1-step RT-qPCR Master Mix (Thermo Fisher Scientific) according to the CDC 2019-nCoV Real-Time RT-PCR diagnostic panel instructions for use (19). This protocol uses three probes: N1, N2, and RP. The samples were considered as positive when all three probes crossed the threshold line within 40 cycles, and were considered as negatives when only RP crossed the threshold line within 40 cycles. The Non Template Controls (NTC) were nuclease-free water and the positive controls were samples with confirmed coronavirus infection.

Samples with Ct value ≤35 were selected for sequencing and viral genomic libraries were constructed using Illumina COVIDSeq Test (Illumina) and checked for quality using 2,200 TapeStation (Agilent Technologies). Libraries were sequenced on NextSeq 500 Sequencing System (Illumina) using NextSeq 500/550 Mid Output Kit v2.5 (300 cycles - Illumina).

### 2.3. Bioinformatic analysis and statistical methods

The quality treatment, mapping, assembly, and variants identifications were performed using the PipeCoV pipeline (20). First, FASTQ files were trimmed using the Phred score (−q 20) as parameters with small reads discarded (−l 20). Later, high-quality reads were mapped to the reference sequence EPI_ISL_402124 (hCoV-19/Wuhan/WIV04/2019) available in the EpiCoV database in GISAID^3^ and the assembly was performed with a kmer size (−k 31). Pangolin v2.3.8 performed lineage identification with the default parameters. Clinical characteristics were analyzed using the Chi-squared test for categorical variables and the Kruskal-Wallis test with correction for multiple comparisons for continuous variables (age). The normality of the dataset was assessed using the Shapiro–Wilk test. All graphs and statistical analyzes were made using R (v.4.2.1). *p*-values <0.05 were considered statistically significant.

### 2.4. Data sources

In addition to our data, we obtained *SARS-CoV-2* genomic, epidemiological, and population information from Brazil and from all continents. The databases used were FIOCRUZ^4^ for all Brazilian regions information and GISAID^3^ for information about all continents. These data from FIOCRUZ and GISAID were collected on November 2^nd^, 2022 and December 3^rd^, 2022, respectively, concerning data from November 2020 to September 2022 for FIOCRUZ and from October 2020 to November 2022 for GISAID. No ethical board approval was requested, given that these databases are public and anonymous.

## 3. Results

We analyzed 1,003 *SARS-CoV-2* genomes obtained from positive COVID-19 patients from Pará (Brazil), divided into three groups according to their clinical symptoms: asymptomatic, mild, and severe (Table 1). Among our samples, 496 (49.45%) were derived from female participants and 507 (50.55%) from male participants, and the average age was 43 years old.

Of the 1,003 samples sequenced, none were infected with Alpha, and two (0.19%) were infected with the Beta variant. The Gamma variant presented the most significant number of cases with 290 (28.91%) cases, followed by Delta with 53 (5.28%). Plus, we found seven (0.69%) Omicron samples in our internal data and 651 (64.9%) non-VOC cases.

Concerning the clinical conditions, 122 (26.70%) of the participants were asymptomatic, 219 (47.92%) had mild symptoms, and 116 (25.38%) had severe disease. A significant association was observed between gender and the clinical condition (female value of *p* = 8.65e-08; male value of *p* = 0.008961) and age (value of *p* = 3.6e-10; Supplementary Figure S2). Regarding variants, Delta (value of *p* <0.01), Omicron (value of *p* <0.01), and non-VOCs (value of *p* <0.01) were significantly associated with the clinical groups.

**Figure 1:**
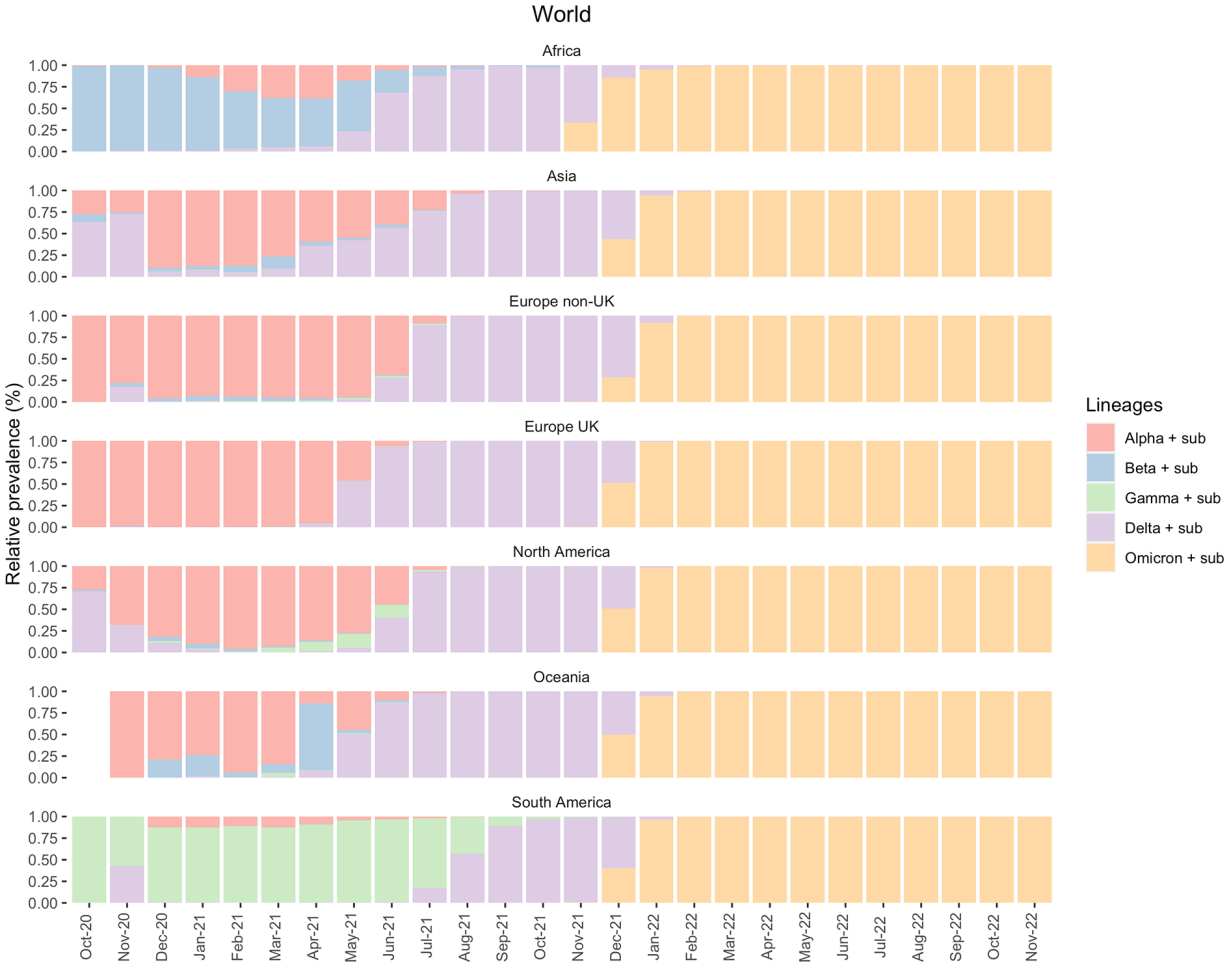
Prevalence of the VOCs and their sub-variants in all continents between October 2020 and November 2022, according to the GISAID database.

We analyzed the distribution of *SARS-CoV-2* variants within a specific time interval in all continents, Brazil and the state of Pará. On a global scale, according to the data deposited in GISAID, until October 2021 the proportion of different variants alters between the continents along the time scale. For instance, until May 2021, Alpha and Beta variants presented a major epidemiological prevalence in Africa, Asia, Europe, North America, and Oceania but not in South America (Figure 1). This dynamic is also observed between June and August 2021, whereas Delta is the dominant variant in all continents except South America. From September, however, the lineages’ pattern is the same across continents, with Delta being the most prevalent between September and December 2021 and Omicron between January 2022 and now (January 2023).

In South America, Gamma was the variant responsible for most cases during the first semester of 2021. As mentioned above, the prevalence of Delta cases was only observed months after its emergence and dominance in other continents. However, from August 2021, the Delta variant modified the epidemiological scenario. It became the most prevalent variant just when the Gamma variant started to disappear until the emergence of Omicron in December 2021 (Figure 1).

**Figure 2:**
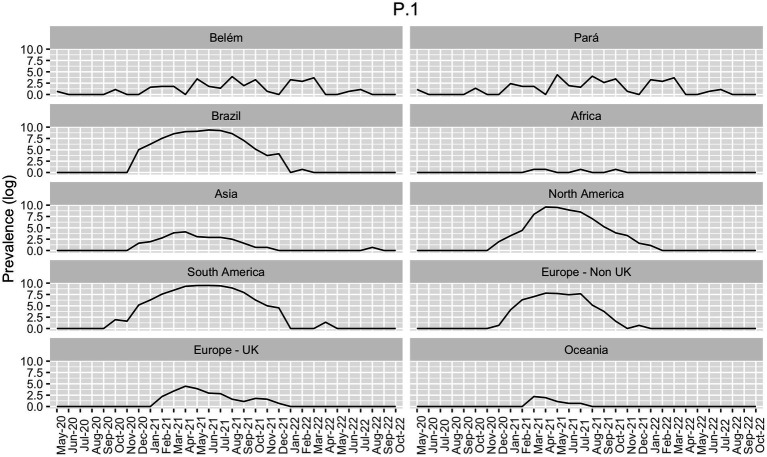
Prevalence of Gamma-like variants in all continents, in Brazil (both from GISAID), and in the state of Pará and in its capital, Belém (own data), in the period of May 2020 to October 2022. Data is presented as the log of the absolute prevalence count.

Brazil’s epidemiological scenario can vastly modify the scenario displayed in South America, given its large population size, as shown in Figure 2. Other South American countries also suffered from an increase in the number of cases after Gamma, such as Argentina, Colombia, Venezuela and Bolivia (21).

**Figure 3:**
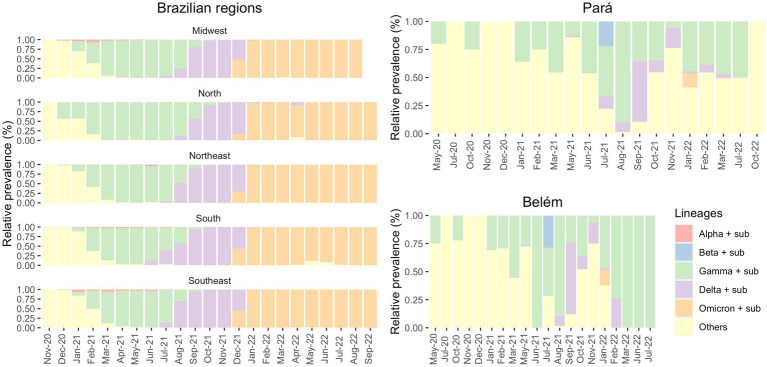
Prevalence of the VOCs and its sub-variants in the Brazilian regions (from GISAID) and in the state of Pará and in its capital, Belém (own data), in the period of May 2020 to October 2022.

In Figure 3, on the other hand, we showed that the period between November 2020 and January 2021 in Brazil was marked by the prevalence of non-VOC in all five Brazilian regions (North, Northeast, Midwest, South and Southeast) (Supplementary Figure S3). However, as 2021 started, Gamma was responsible for most COVID-19 countrywide cases between January 2021 and July 2021, followed by Delta in August 2021 and Omicron in January 2022.

It is worth mentioning that there are huge differences between the Brazilian regions. The North region has several differences compared to the other regions of Brazil, like socioeconomic, cultural and health dissemblances. Usually, the Southeast region has more financing, especially due to its large concentration of people. These differences between the regions were also demonstrated in the pandemic given that some regions were financially poorly assisted to combat COVID-19, while others had enough fundings. Another example of these variations is the fact that quite a few Alpha cases were observed in the North, where Gamma prevailed for the longest time (April–September 2021).

Figure 3 shows the distribution of *SARS-CoV-2* variants. Observing the Brazilian regions, we can see that Gamma (in green) was the most prevalent variant for an extended period rather than Alpha and Beta (which were the most prevalent in other countries), especially in northern Brazil. Furthermore, we observed that in Belém, until May 2021, non-VOC were the most dominant variants. This demonstrates a greater diversity of circulating variants that diminished with the increase of Gamma cases from June 2021. In addition, according to FIOCRUZ data, P.2 variant had its greater transmission between December 2020 and February 2021, while Gamma (P.1) presented higher prevalence in Pará state between April 2021 and September 2021.

**Figure 4:**
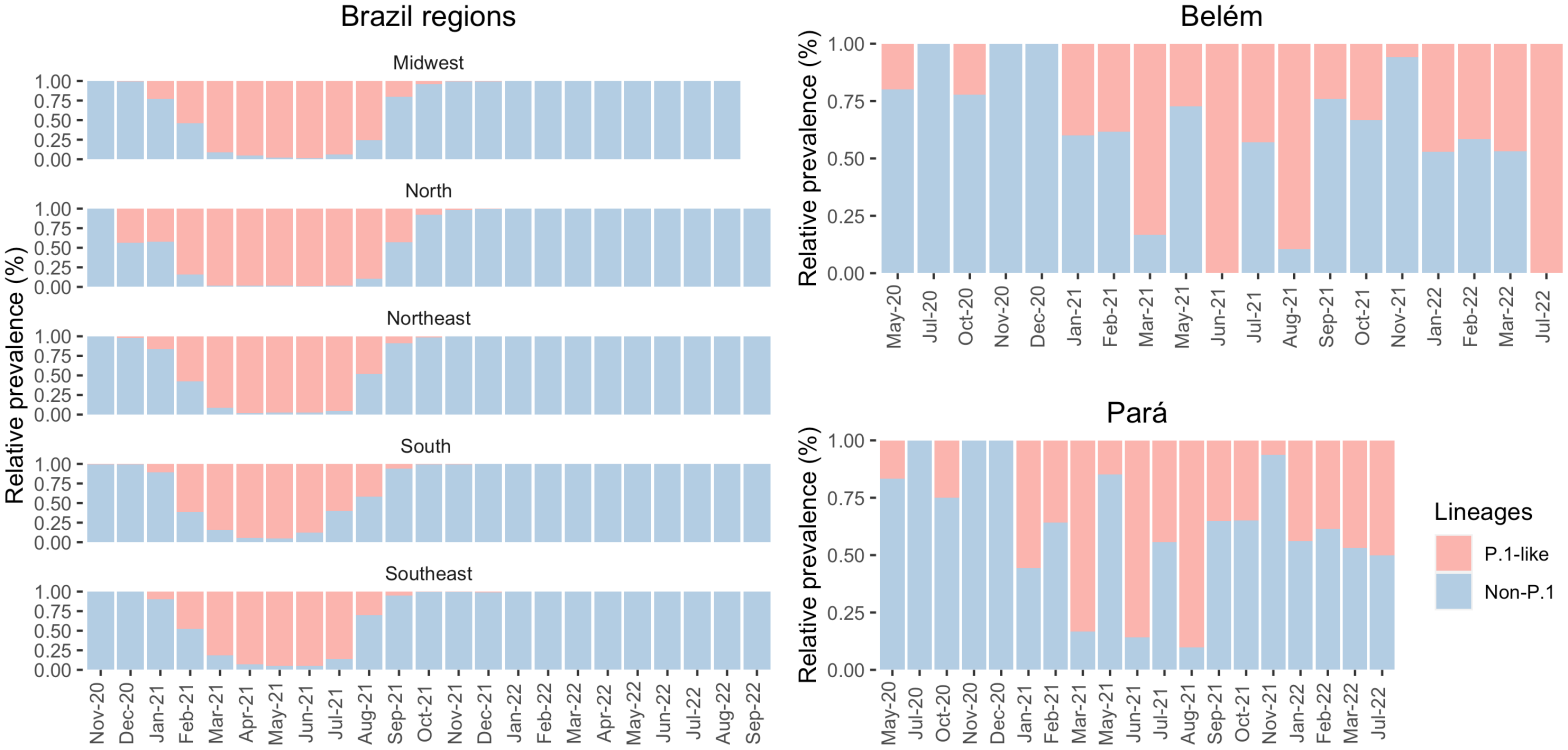
Prevalence of P1-like and non-Gamma variants in the Brazilian regions (from GISAID) and in the state of Pará and in its capital, Belém (own data), in the period of May 2020 to September 2022.

Our data showed a few cases of Gamma infections in May 2020 and October 2020 in Belém (Figure 4). Therefore, we decided to compare the distribution of the Gamma variantin Brazil. As shown in Figure 2, Gamma caused a new wave of infections between January and August 2021 in Brazil, with cases until September in Midwest and North regions. However, according to our own dataset, in both Pará and Belém, the first case of Gamma infection was reported in May 2020, and it persisted with high prevalence until 2022. In Pará, Gamma may have prevented a wave of infections by the Delta variant in the state, by dominating the area before Delta arrived.

When comparing Brazil, Pará, and Belém with the continents between May 2020 and October 2022, it becomes clear that Brazil’s epidemiological status has molded the South Americas’ given that their Gamma prevalence patterns are very similar (Figure 2). In parallel, the Brazilian North region had an important role in the maintenance of the Gamma variant in Brazil. In addition, there was a lower prevalence of Gamma in Asia, Africa, Europe, and Oceania, indicating that this variant did not present dissemination success in these continents. On the other hand, the Delta variant has emerged in Asia and has fastly disseminated across those continents (Figure 2).

## 4. Discussion

The State of Pará is located in the Brazilian Amazon and is the second largest state in Brazil, with a population of more than 8.7 million inhabitants. Belém is the capital and has about 1.5 million inhabitants (22). Pará comprises 144 municipalities, and we obtained 1,003 samples from 67 of them (Supplementary Figure S1).

Among these samples, we found a significant association between sex and clinical condition (female value of *p* = 8.65e-08; male value of *p* = 0.008961) and age and clinical condition (value of *p* = 3.6e-10). Delta, Omicron, and the non-VOC presented significant associations with clinical conditions. In contrast, despite being the most incident VOC, Gamma did not show any significant association with the clinical condition in our analysis. The P.1-like variants demonstrated massive participation in the Pará and Belém infections (Figure 2), appearing even when Delta and Omicron dominated the national epidemiological scenario, despite not having influenced the proportion of deaths caused by COVID-19 in the first (7.7%) and second wave (2.3%) in Pará However, according to Freitas et al. (23), Gamma may be related to the increase in deaths between young (20 to 59 years) and female patients in the State of Amazonas. They showed that the proportion of deaths in female patients increased from 34% in the first wave to 47% in the second wave of COVID-19 and that the number of hospitalizations between young patients was approximately 2.7 times higher in the second wave in comparison to the first one. Gamma also presented an association with the increase in deaths and severe cases among patients without previous comorbidities - it increased from 31 to 50% in the first to the second wave, respectively, after the emergence of the Gamma variant (23). These differences in the outcome between these two states may be explained by the higher incidence of Gamma in Amazonas since it emerged there and by the health system collapse.

According to FIOCRUZ data, in Brazil the first wave of COVID-19 started in April 2020, and the second wave in December 2020, this last one strongly influenced by the increasing Gamma variant cases (16). It is important to highlight that during the first wave, we observed a greater diversity of circulating variants worldwide and in Brazil. However, after the second wave, the epidemiological scenario was dominated by Delta, and Omicron in December 2020, July 2021, and December 2021, respectively (24). Data from FIOCRUZ showed that the Gamma variant was the first VOC to vastly dominate the epidemiological scenario in all Brazilian regions from 2020 until July 2021 (16). In the second semester of 2021, Delta caused a new wave of infections and hospitalizations that was followed by Omicron and its sub-variants (such as BA.1, BA.2, XBB.1., BQ., and FE) in December 2021, which quickly became the most detected strains in the country, reaching almost 100% of COVID-19 cases until May 2023 (25).

In contrast, our data has shown another pattern: non-VOC were responsible for most of the cases at the beginning of the pandemic. From December 2020/January 2021 onwards, Gamma became the most prevalent variant, followed by Delta and Omicron. Gamma (P.1) caused severe public health problems in the North region of Brazil as it was decisive in increasing the number of cases and death of COVID-19. This variant emerged in Amazonas (AM), a neighboring state of Pará (Figure 3), and presents a high transmission capacity and virulence (17, 26).

Gamma reached its highest level of transmission between January and June 2021, a period that coincides with the second wave of COVID-19 and with the highest number of hospitalizations and deaths caused by the coronavirus in Amazonas, Pará, in the rest of Brazil and in South America (27–29). According to De Souza et al., there was an increase to almost 3,000 daily deaths and hospitalizations during the second wave in Amazonas caused mostly by the P.1 variant (30). At this time, the Brazilian National Public Health System (SUS) almost collapsed - the occupancy rate of ICU (Intensive Care Unit) was 90%, leading to the most severe health crisis ever experienced in Brazil (31). Therefore, Gamma had a central role in the vast proportion of COVID-19 during its second wave in Brazil and South America since Brazil represented more than 55% of total cases and deaths from this continent (32). However, it is essential to highlight that Gamma infection itself may not have been the sole cause of this high mortality rate - other factors may have influenced it, such as the shortage of respiratory equipment and intensive care units.

In the global scenario, the emergence of the Gamma variant coincided with a transient increase in the number of cases and deaths, especially in January and February 2021. As shown in Figure 2, in 2021 this VOC did not have significant incidence in countries outside the Americas as it had in Brazil and in other countries such as Mexico and Bolivia with Gamma sub variants, like P.1.7.1 and P.1.10.2 (33). In this context, (33) reported that, between June and August 2021, Gamma variant stood out in Argentina. However, according to Bastos and colleagues, the Brazilian second wave significantly increased severe COVID-19 cases in Africa and the UK (34). Soon after, the number of reported cases rapidly decreased and increased in late March 2021, when Delta began to dominate COVID-19 cases worldwide (24).

The first cases of Gamma were officially detected in January 2021 (35, 36), but there is evidence of this variant in the state of Amazonas before the mentioned date. A few days later, the Instituto Leônidas & Maria Deane (ILMD/FIOCRUZ Amazônia), in collaboration with Fundação de Vigilância em Saúde do Amazonas (FVS-AM), released a technical note stating that Gamma would be derived from B.1.1.28. This note also declared that in December 2020, about 51% of the SARS-CoV-2 genomes in Amazonas were Gamma — this number increased to 91% in January 2021 (27). Although Gamma was officially identified in December 2020/January 2021 (35, 36), there were four sequenced samples from Belém, one in May 2020 and three in October 2020, that were infected with Gamma-like variants, according to Pangolin, UShER and Scorpion databases. To the best of our knowledge, there are no studies before 2021 that discuss the transmission of Gamma-like variants in Brazil. Therefore, this fact may indicate that this variant emerged in northern Brazil several months before its identification and that Gamma and its sub variants (like P.1.4, P.1.7, P.4, and others) demonstrated their actual transmission capacity only at the end of 2020 The significant prevalence of Gamma in the North of Brazil may have delayed the entry of the Delta variant in the region. According to Figure 4, Gamma represented most cases in the Southeast region between March and July 2021 and in the North region between February and August 2021. Meanwhile, Delta was detected in the Southeast region in July 2021 and prevailed over the other VOCs from August to December 2021. In the North region, Delta appeared in August 2021 and presented the highest number of cases only from September 2021 until December 2021. It demonstrates that Gamma dominated the prevalence scores for a longer period, which may explain the late detection of Delta in the North region of Brazil (37).

In these pandemic times, next-generation sequencing has proven to be extremely useful in the fight against SARS-CoV-2 - it allows real-time identification of the virus variants. It provided strong evidence about its transmission dynamics. This genetic and epidemiological information is precious to direct sequenced-based public health surveillance and can be applied against other infectious diseases, such as chikungunya and malaria (38). Brazil’s zoonotic pathogens sequencing and genomic surveillance capacity are conducted by only a few research institutions and universities capable of supporting Brazilian regions, using different types of sequencing technologies. So, a significant investment in genomic capacity is critical to empower surveillance in Brazil and would vastly improve global efforts to combat this pandemic and any new emergent pathogen.

## 5. Conclusion

This study shows the importance of investigating the SARS-CoV-2 transmission dynamics to understand the different prevalence patterns of its variants across global regions. Here, we focused especially on the state of Pará, in the North of Brazil. Our results show that Brazil represented a unique epidemiological status since it was where Gamma (P.1) emerged This variant had a higher transmission ability and presented an increased prevalence in South America compared to the rest of the world, especially during 2021 as seen in Figures 1, 2. From our sequencing, we also suggest an early case of Gamma-like infection in the state of Pará, indicating that this VOC may have appeared a few months before its formal identification. It reinforces the relevance of having a robust genomic surveillance service as it allows better management of the pandemic and efficient solutions to possible new disease-causing agents, like viruses, bacteria, and fungi.

## Data availability statement

The datasets presented in this study can be found in online repositories. The names of the repository/repositories and accession number(s) can be found in the article/Supplementary material.

## Ethics statement

The studies involving human participants were reviewed and approved by 50865721.1.0000.0017. The patients/participants provided their written informed consent to participate in this study.

## Author contributions

CP and AV: writing—original draft preparation. TN, RO, JA-P, GS, and GN: bioinformatic analysis. CP, AV, MB, LC, JP, CB-d-S, CS, LM, and PP: sample processing and sequencing. AV: statistical analysis. JV, RB, MSS, MBS, PFC, CGS, and ÂR-d-S: sample acquisition. WD and GO: funding. GO: study conception and supervision. All authors have read and agreed to the published version of the manuscript.

## Funding

This work was funded by Vale (COVID-19, RBRS000603.12) in collaboration with Fiocruz to support the Research Network for the Genomic Sequencing of *SARS-CoV-2*. By Fundação de Amparo a Estudos e Pesquisas no Estado do Pará (FAPESPA) to support the COVID-19 Genomic Surveillance Network based on New Strategies for Diagnosis, Prevention, and Prognosis (Rede de Vigilância Genômica da COVID-19 baseada em Novas Estratégias de Diagnóstico, Prevenção e Prognóstico).

## Conflict of interest

The authors declare that the research was conducted in the absence of any commercial or financial relationships that could be construed as a potential conflict of interest.

## Publisher’s note

All claims expressed in this article are solely those of the authors and do not necessarily represent those of their affiliated organizations, or those of the publisher, the editors and the reviewers. Any product that may be evaluated in this article, or claim that may be made by its manufacturer, is not guaranteed or endorsed by the publisher.

## Footnotes

^1^ https://ourworldindata.org/coronavirus

^2^ https://www.itv.org/imprensa/projeto-genoma-covid-19/

^3^ https://www.gisaid.org/

^4^ http://www.genomahcov.fiocruz.br/dashboard/
