# Supplementary figures and images for: Transmission dynamics of SARS-CoV-2 variants in the Brazilian state of Pará
Source: Front Public Health. 2023 Jul 5;11:1186463. doi: 10.3389/fpubh.2023.1186463 (PMC10543262; doi:10.3389/fpubh.2023.1186463)

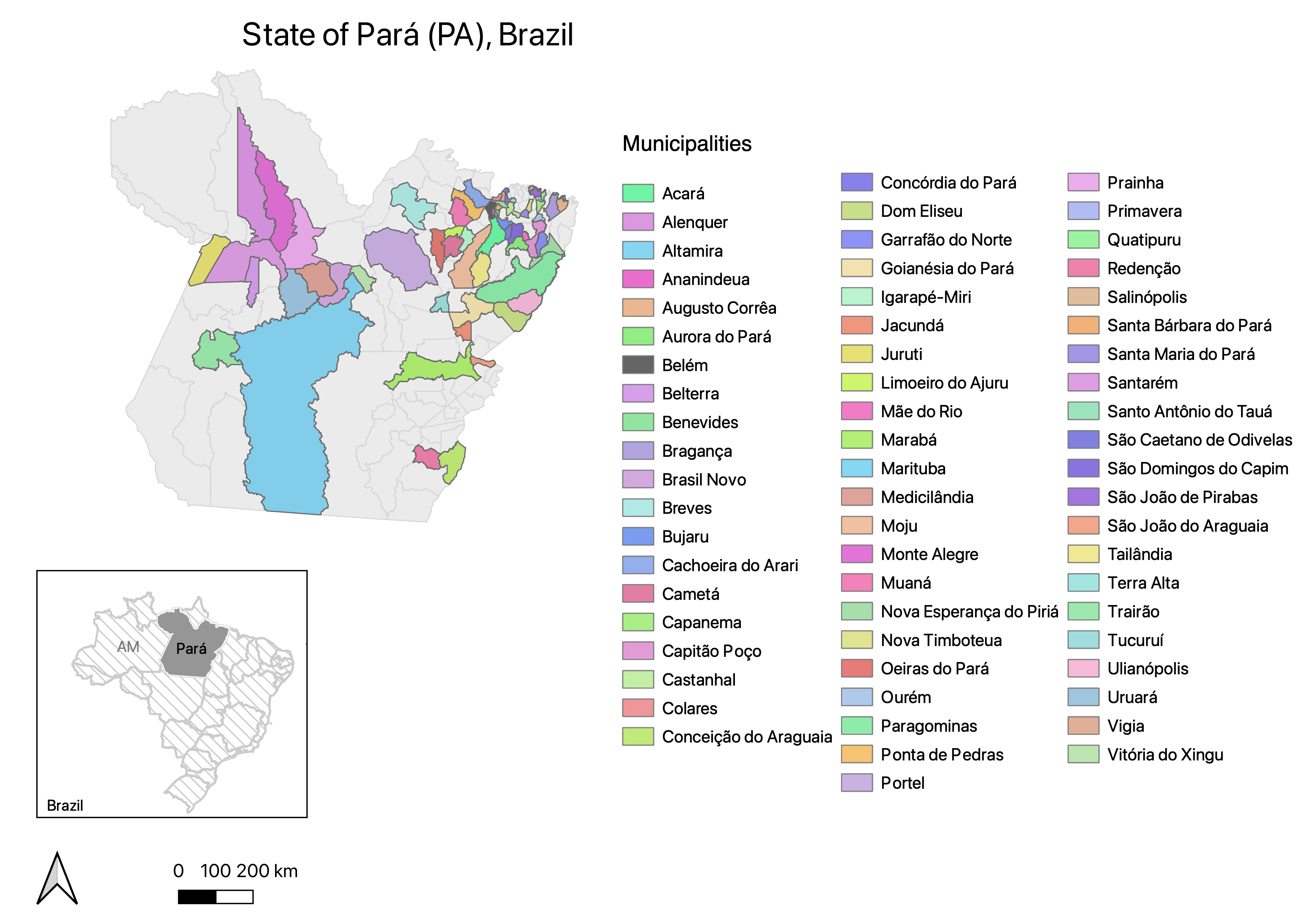

Supplement: Supplementary file 2 [file Image_1.tiff]

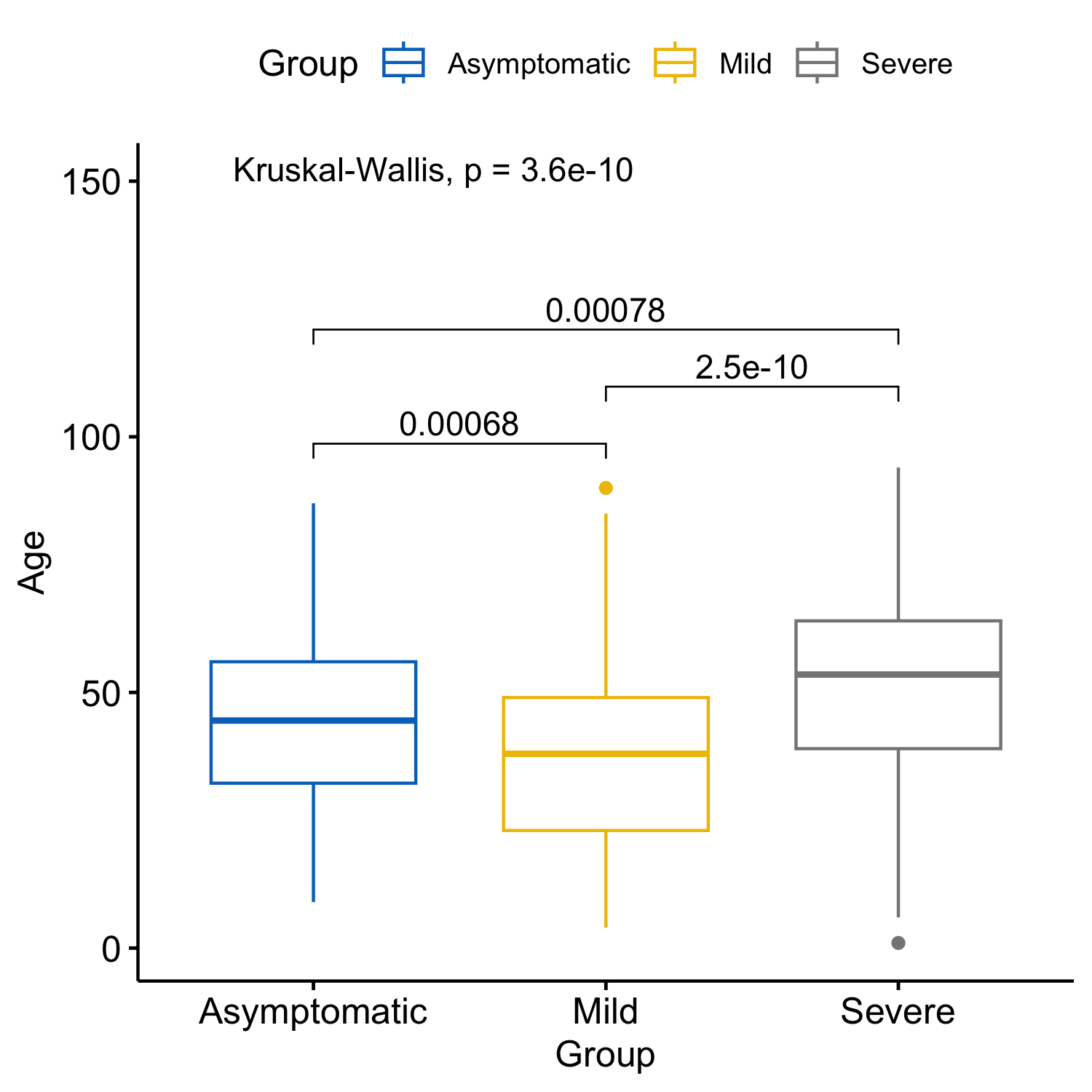

Supplement: Supplementary file 3 [file Image_2.tiff]

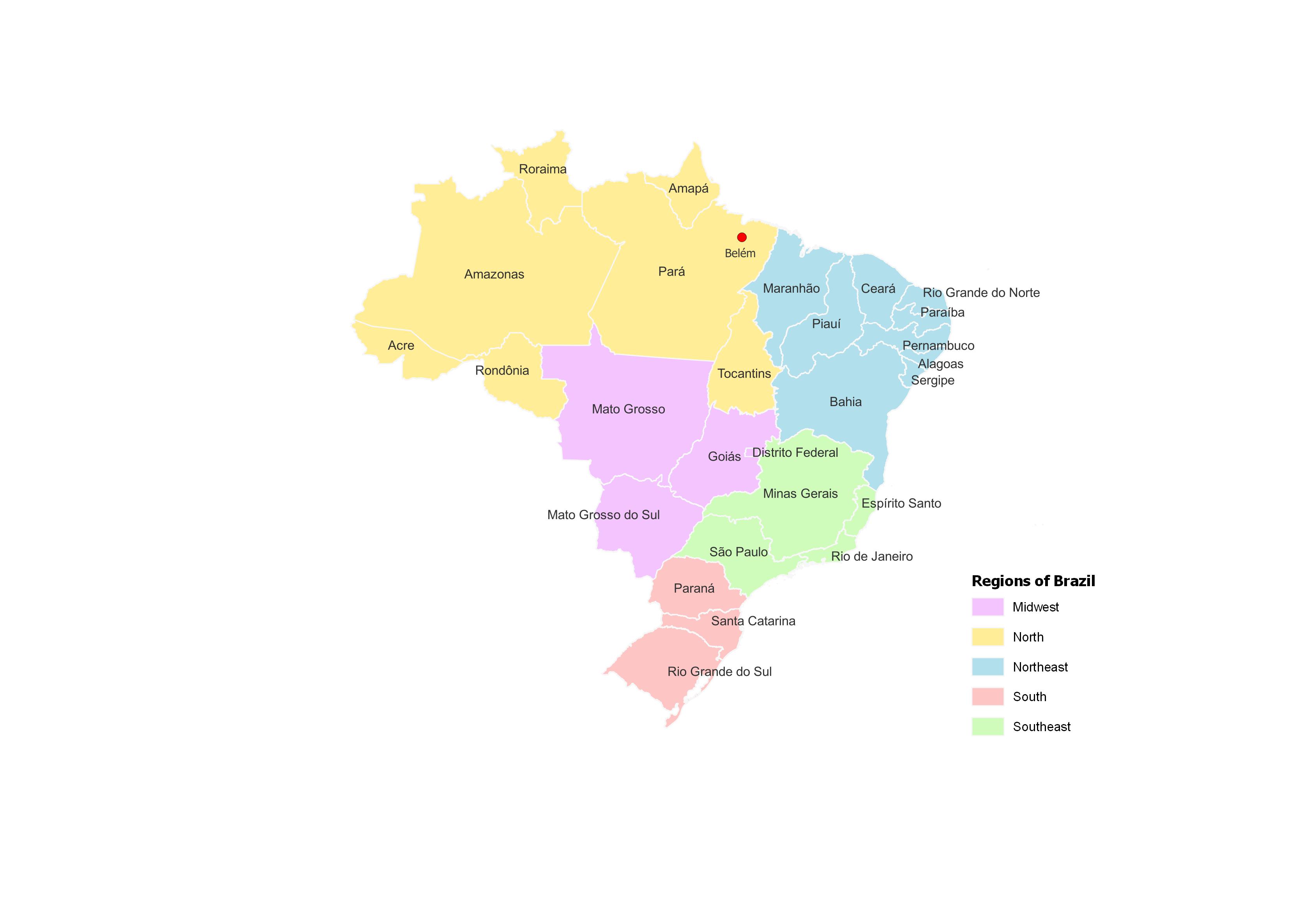

Supplement: Supplementary file 4 [file Image_3.jpeg]
